# Stepwise Reduction of Ruthenium and Growth of Magnetic Structure During Hydrothermal Crystallisation of SrRu_2_O_6_ from KRuO_4_

**DOI:** 10.1002/anie.202521810

**Published:** 2025-11-17

**Authors:** Mark Crossman, Craig I. Hiley, Helen Y. Playford, Chris M. Goodway, Ronald I. Smith, Thomas C. Hansen, Richard I. Walton

**Affiliations:** ^1^ Department of Chemistry University of Warwick Gibbet Hill Road Coventry CV4 7AL UK; ^2^ ISIS Neutron and Muon Facility Rutherford Appleton Laboratory Didcot OX11 0QX UK; ^3^ Institut Laue‐Langevin 71 Avenue des Martyrs CS 20156, 38042 Grenoble Cedex 9 France

**Keywords:** Antiferromagnetism, Crystallisation, Hydrothermal, Neutron diffraction, Ruthenium

## Abstract

Using a new design of hydrothermal reactor constructed from a null‐scattering TiZr alloy in situ powder neutron diffraction data are collected to follow the crystallisation of the Ru(V)‐containing high temperature antiferromagnet SrRu_2_O_6_. Full pattern fitting allows quantitative analysis of the evolution of crystalline phases during reaction, revealing the decay of the Ru(VII) precursor KRuO_4_, the transient presence of the Ru(VI) oxyhydroxide SrRuO_3_(OD)_2_ and the eventual growth of SrRu_2_O_6_. At 140 °C crystallisation is retarded, prolonging the lifetime of SrRuO_3_(OD)_2,_ while at 180 °C complete formation of SrRu_2_O_6_ is observed. At the same time, evolution of the magnetic Bragg peak intensities resulting from the antiferromagnetic ordering of Ru(V) moments in SrRu_2_O_6_ is seen. This unique observation is possible since crystallisation occurs below the Néel temperature of the material. The magnetic structure is found to grow concurrently with the atomic structure, with calculated magnetic moment per Ru consistent with ex situ studies at the same temperatures. The reported experiment design will be applicable for the study of many other families of inorganic materials that crystallise under solvothermal conditions.

## Graphical abstract

In situ *n*eutron powder diffraction data collected while heating the Ru(VII) reagent KRuO_4_ in the presence of aqueous Sr^2+^ reveal the crystallisation of a Ru(VI) oxyhydroxide before the expected Ru(V) oxide; at the same time evolution of antiferromagnetism in the final SrRu_2_O_6_ product follows the growth of its crystal structure.

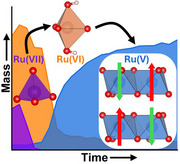

## Introduction

The use of hydrothermal crystallisation as a synthesis method for multinary transition‐metal oxide materials was originally established for the perovskite BaTiO_3_ in the 1950s,^[^ ^1^ ^]^ and now provides a route to various perovskites that incorporate metal cations from all parts of the Periodic Table, including magnetic materials such as manganites, multiferroic materials such as chromites and ferroelectric niobates and zirconates.^[^ ^2^ ^]^ In the past decades hydrothermal chemistry has been more widely extended to provide a versatile preparative method for numerous mixed‐metal oxides that have interesting, and useful, properties; including fluorites, pyrochlores, rock‐salts,^[^ ^3^, ^4^, ^5^, ^6^, ^7^, ^8^ ^]^ as well as mineral analogues with more complex structures and compositions.^[^ ^9^ ^]^ The advantages of the mild reaction conditions for these materials that might otherwise be prepared using traditional solid‐state chemistry lie in the formation of fine powders of materials (nanoscale to microscale) with the potential to control crystal morphology and form that might allow processing for practical applications or tuning of properties. From the point of view of synthesis, the attraction of the use of hydrothermal preparation of oxides is the possibility of crystallisation of compositions and crystal structures not accessible by other methods.^[^ ^10^ ^]^

Particularly illustrative of the role of hydrothermal synthesis in preparative materials chemistry is a growing family of alkali‐earth ruthenium oxides and oxyhydroxides that crystallise directly from water at ∼200 °C. Examples include SrRu_2_O_6_,^[^ ^11^, ^12^ ^]^ and an analogue BaRu_2_O_6_,^[^ ^13^ ^]^ that have related honeycomb layered structures and both showing unusually high magnetic ordering temperatures, and the hexagonal 8H‐perovskite Ba_4_Ru_3_O_10.2_(OH)_1.8_.^[^ ^14^ ^]^ In total at least eight distinct ruthenates have been reported by this method, and these materials, which typically contain Ru(V), are all prepared using the ruthenium(VII) reagent KRuO_4_ which when heated in water with alkali‐earth metal salts yields the ternary phases directly.^[^ ^11^, ^15^ ^]^ The exotic magnetic and electronic properties of alkali‐earth ruthenates,^[^ ^16^ ^]^ as well as their use in catalysis,^[^ ^17^ ^]^ continue to attract attention, and so it would be of clear benefit to understand the crystallisation of these materials with a greater level of detail. With knowledge of the pathways involved in the assembly of materials under reaction conditions, including their kinetics of crystallisation and the competitive formation of distinct phases, planning the synthesis of new materials with desired structural features for properties would be possible, as well as being streamlined and optimised for efficient preparation for applications.

A large number of X‐ray scattering methods have now been developed for following crystallisation of inorganic materials in situ under hydrothermal conditions,^[^ ^18^, ^19^, ^20^, ^21^, ^22^ ^]^ including in laboratory‐scale steel autoclave reactors,^[^ ^23^, ^24^, ^25^, ^26^ ^]^ and in capillary devices,^[^ ^27^, ^28^, ^29^, ^30^ ^]^ where the latter design also allows total scattering measurements, with both Bragg diffraction and total scattering for pair distribution function analysis measured in real time to examine local as well as long‐range order.^[^ ^31^ ^]^ In this current work we develop the use of neutron diffraction to follow hydrothermal crystallisations, which has so far has been reported only relatively few times,^[^ ^32^, ^33^, ^34^, ^35^, ^36^, ^37^, ^38^, ^39^, ^40^, ^41^, ^42^ ^]^ compared to X‐ray diffraction. As well as the highly penetrating nature of neutrons, their use offers the distinct advantage of designing “null scattering” reaction cells that give minimal background signal, such that cells with volume similar to laboratory vessels can be constructed that give no sharp diffraction features and minimal background. Of relevance for the system we study here, the observation of magnetic as well as nuclear scattering is a further advantage of applying neutron scattering. With the availability of high‐flux neutron sources and high‐count‐rate instrumentation, our work showcases the use of neutron scattering for in situ studies of crystallisation under realistic reaction conditions. We study the crystallisation of SrRu_2_O_6_ from water to provide unprecedented detail of the evolution of crystallinity and magnetic structure during the formation of an oxide under hydrothermal conditions. This particular material has been the focus of much interest, both experimental and theoretical, because of its unusual magnetic properties^[^ ^43^ ^]^ that have potential applications in spintronics.^[^ ^44^ ^]^

## Results and Discussion

**Figure 1:**
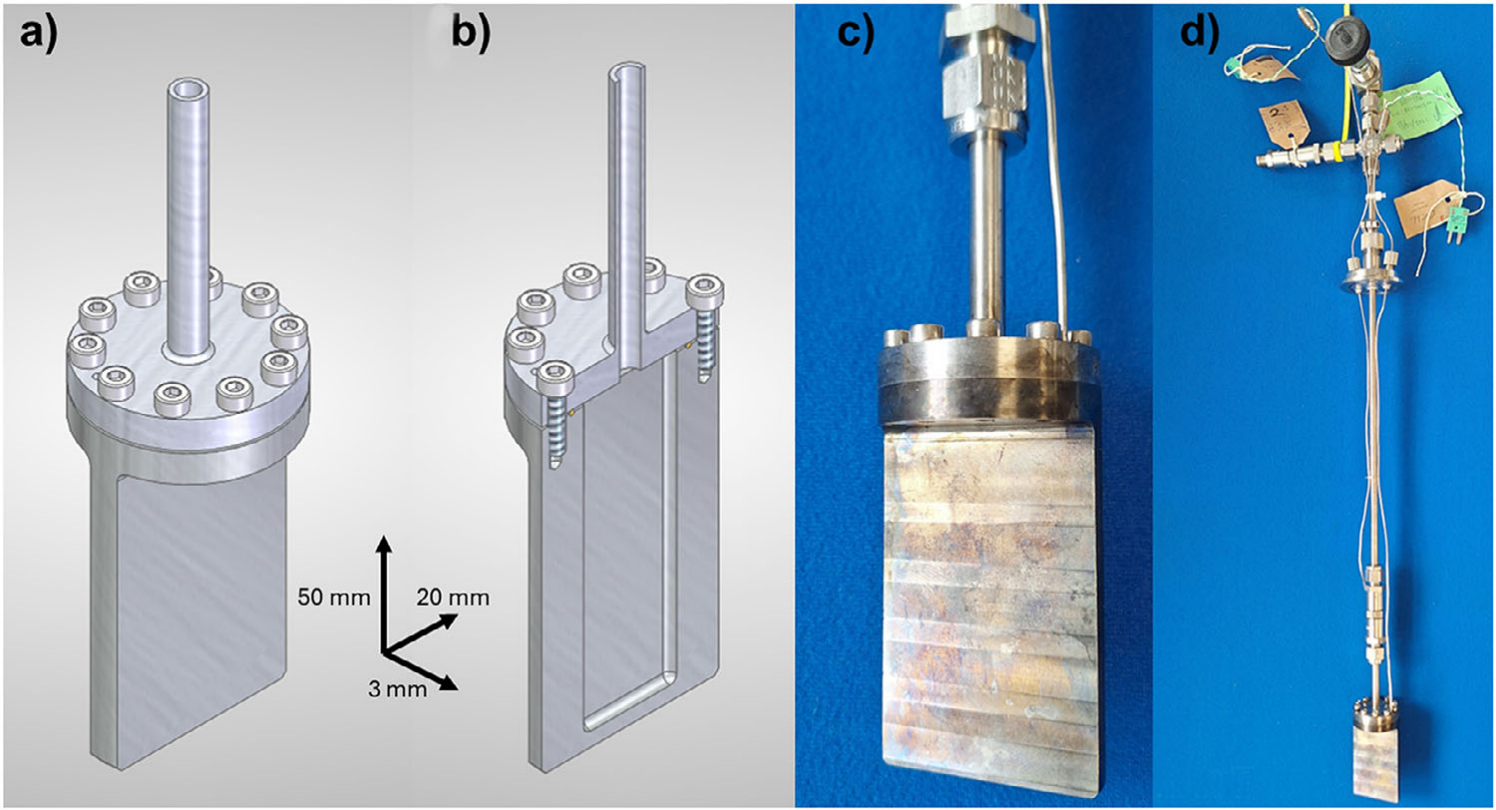
Images of Ti─Zr hydrothermal cell for in situ neutron scattering. a) Schematic of the cell with dimensions indicated (note these are internal measurements of the sample chamber). b) Cutaway view showing internal reaction chamber, c) photograph of cell and d) photograph of assembly of the cell and pressure relief system. Further images of the cell assembly are provided in Figure S1.

Figure 1 shows the design of the in situ hydrothermal cell used in this work. The main cell body is a thin rectangular slab in shape constructed entirely from a single machined block of null‐scattering Ti─Zr alloy, Ti_62_Zr_38_,^[^ ^45^ ^]^ with a total internal volume of 3.3 mL. Powder neutron diffraction measurements were carried out on two instruments at two different facilities. Time‐of‐flight data were collected using Polaris^[^ ^46^ ^]^ at the ISIS Neutron and Muon Facility, UK, while constant wavelength data were collected on the D20 diffractometer at the Institut Laue‐Langevin, France.^[^ ^47^ ^]^

The flat cell design was chosen so that the path of neutrons through large amounts of water was avoided, since earlier cylindrical cell designs, which although more closely mimicking the geometry of laboratory hydrothermal autoclaves, gave rise to significant background signal from the large *Q*‐dependent scattering from the liquid solvent.^[^ ^33^ ^]^ In this new design the entire cell is heated by placing it in a cylindrical geometry furnace (Figure S2) operating in the vacuum of the neutron beamline sample chamber. This means that the whole of the reaction chamber can be exposed to the neutron beam (see Supporting Information) and provides a setup in which no stirring is required to suspended solid powder in the liquid reaction medium: thus even if material settles during chemical reactions scattering from this is also measured. This overcomes earlier design issues of stirred reactors, where the success and consistency of stirring is very difficult to determine once a cell is sealed, and the beam position needed to be above the stirring device to avoid extra, unwanted, background signal.^[^ ^24^, ^33^ ^]^ In fact, the new design presented here more closely replicates most laboratory hydrothermal reactions that are performed under static conditions.

To avoid the incoherent scatter of neutrons by protons, that give rise to large backgrounds in neutron scattering experiments,^[^ ^48^ ^]^ D_2_O was used in place of H_2_O for all in situ crystallisation experiments. Initial experiments were performed in the laboratory to ensure that the synthesis of SrRu_2_O_6_ was reproducible under these conditions, also using a 3 mL Teflon reactor to mimic the small size of the in situ cell, and powder XRD patterns from the resulting solid showed formation of phase‐pure sample of similar crystallinity to material prepared in H_2_O (Figure S3).

**Figure 2:**
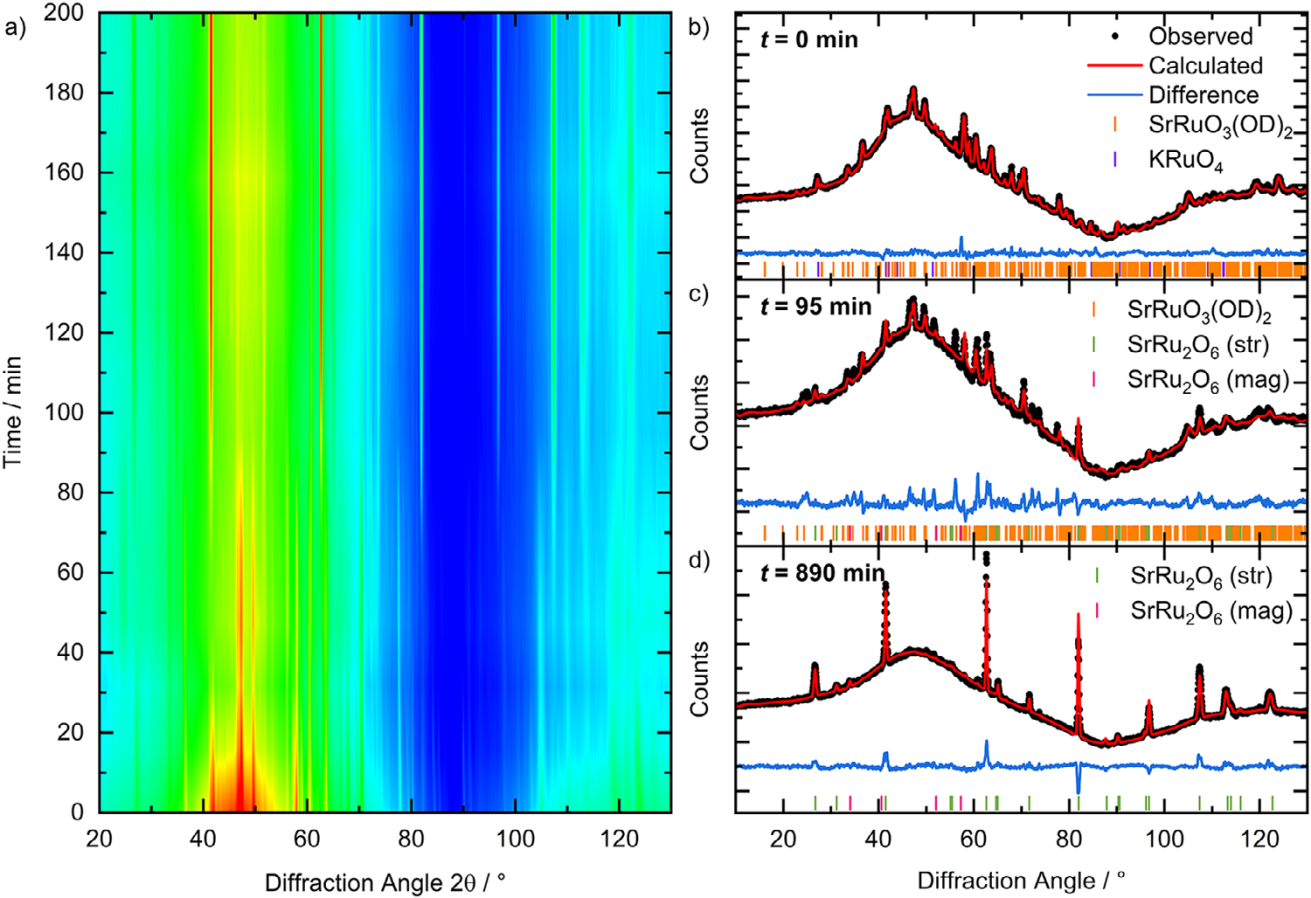
a) Contour colour map of in situ neutron diffraction (D20, ILL *λ* = 2.41 Å) measured at 180 °C from reaction mixture of SrO_2_:2KRuO_4_ in D_2_O from 0 min to 200 min. b)–d) Rietveld fits (using TOPAS software ^[^ ^51^ ^]^) to individual patterns measured at b) the start of the reaction (0 min), c) the point at which the significant amounts of SrRuO_3_(OD)_2_ and SrRu_2_O_6_ are present (95 min) and d) at the end of the reaction (890 min).

Figure 2a shows a contour colour‐map representation of crystallisation at 180 °C from 20 mmol KRuO_4_ and 10 mmol SrO_2_ in 3 ml D_2_O using the D20 diffractometer at the ILL facility. The formation of the strongest Bragg peaks of the product SrRu_2_O_6_ are observed after ∼100 min and these increase in intensity over the following 900 min (not shown in Figure 2a). It is noteworthy that at the initial stage of reaction Bragg diffraction from crystalline material is observed, which evolves upon heating. It can also be noted that the background signal changes, which may be indicative of the solution structure and/or composition changing as materials crystallise from it. In our previous work we found that after short reaction times or at moderate reaction temperature (100 °C) the Ru(VI) oxyhydroxide SrRuO_3_(OH)_2_ crystallises under the same reaction conditions as used here,^[^ ^49^ ^]^ making this an obvious candidate for the transient phase seen in situ. To analyse the in situ crystallisation data we thus used the published structure of KRuO_4_^[^ ^50^ ^]^ and the structure of SrRuO_3_(OH)_2_ obtained previously (with H replaced by D),^[^ ^49^ ^]^ with the goal of quantifying the growth and decay of the transient phase and its influence on the formation of the final product. A multiphase pattern fitting of each diffraction pattern collected in situ was undertaken, and Figure 2b–d show typical Rietveld fits obtained at distinct stages of the reaction at 180 °C. At intermediate reaction times a two‐phase mixture of SrRuO_3_(OH)_2_ and SrRu_2_O_6_ gave the best fit to the data, but there are some minor unindexed Bragg peaks (for example at 2*θ* ∼ 55° on Figure 2c): these could not be matched to any known strontium ruthenate and we were unable to isolate any extra phase by quenching. Analysis of the final diffraction pattern, Figure 2d, shows the presence of only SrRu_2_O_6_, noting that peak intensities are slightly affected by the non‐standard geometry of our reaction cell.

**Figure 3:**
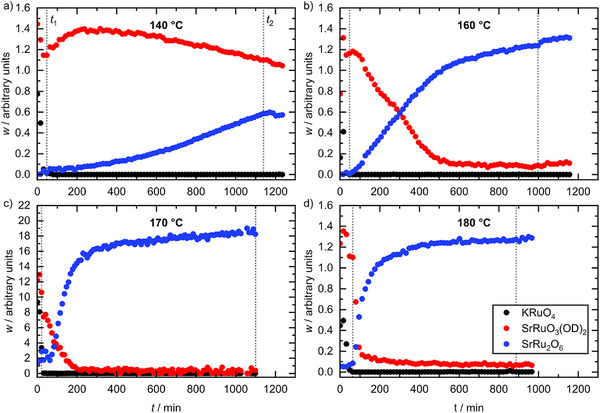
Crystallisation curves obtained at a) 140 °C, b) 160 °C, c) 170 °C, and d) 180 °C. Vertical lines indicate *t*_1_ (when the cell reached reaction temperature) and *t*_2_ (when the cell heating ceased and the cell was allowed to begin cooling. Uncertainties (one estimated standard deviation (1*σ*)) in *w* are smaller than data points. Note that data in c) were from a time‐of‐flight experiment, and the difference in the overall magnitude of *w* is due to different data normalisation procedures, but the cell and reaction conditions were the same.

Two further reaction temperatures were studied using the D20 diffractometer, 140 °C and 160 °C (Figure S4). A further experiment was carried out on the Polaris diffractometer at the ISIS facility at 170 °C (Figures S5–S8). The aim here was to collect data using two diffraction configurations (time‐of‐flight (fixed detector) versus fixed wavelength (angle‐dispersive)) to show the versatility of the cell at different neutron facilities. The refined relative mass (*w*, defined in the Experimental Section) of KRuO_4_, SrRuO_3_(OD)_2_ and SrRu_2_O_6_ as a function of time at the four reaction temperatures studied is shown in Figure 3. Some undissolved precursor KRuO_4_ is seen initially in all experiments but is short‐lived in presence of water and in all cases was completely dissolved/reacted before the cell reached the reaction set temperature. SrRuO_3_(OD)_2_ is then the main phase seen in the initial stages of the reaction before SrRu_2_O_6_ is observed. The decomposition of SrRuO_3_(OD)_2_ into SrRu_2_O_6_ is rather sluggish at 140 °C and was incomplete after 1100 min (Figure 3a), with both SrRuO_3_(OD)_2_ and SrRu_2_O_6_ present in roughly equal proportions. At 160 °C, the reaction was mostly complete after 1000 min, with SrRu_2_O_6_ beginning to form after ∼60 min, and SrRuO_3_(OD)_2_ detected until ∼600 min (Figure 3b). The reactions at 170 °C and 180 °C were notably faster, with all SrRuO_3_(OD)_2_ consumed by 200 min (Figure 3c,d). The amount of SrRu_2_O_6_ continues to increase slowly after SrRuO_3_(OD)_2_ is no longer detected, suggesting that some Sr and Ru remain in solution and gradually crystallise into SrRu_2_O_6_. This would be consistent with the differing Sr:Ru molar ratios in the two materials which would also suggest a dissolution‐recrystallisation process, i.e., that the transient SrRuO_3_(OD)_2_ dissolves to allow crystallisation of SrRu_2_O_6_ leaving excess Sr^2+^ in solution, rather than a solid‐state transformation which would imply maintaining the Sr:Ru molar ratio. This is also consistent with the very different crystal structures of the two materials, with SrRuO_3_(OD)_2_ containing trigonal bipyramidal Ru(VI) with axially coordinated hydroxides, with no connections between Ru centres, while SrRu_2_O_6_ contains octahedral Ru(V) that share mutual edges to create infinite layers. The agreement (*R*_wp_) of the Rietveld fits to the data remains constant for each experimental run (Figure S9).

**Figure 4:**
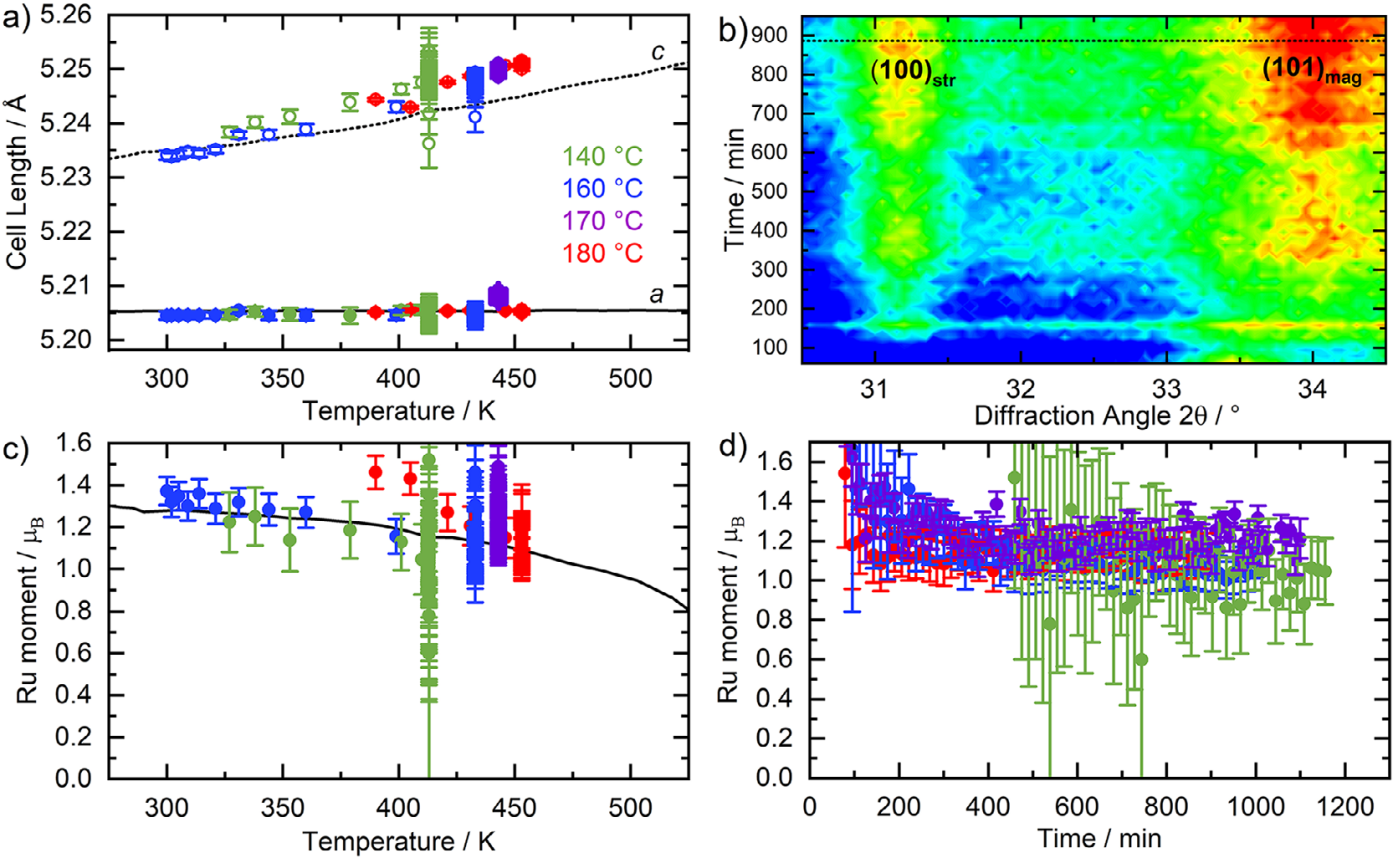
a) *a* (closed symbols) and *c* (open symbols) cell parameters measured in situ, b) contour colour map of in situ neutron diffraction (D20, ILL *λ* = 2.41 Å) measured at 180 °C within the region of the SrRu_2_O_6_ (101) peak for the magnetic unit cell from 60 to 950 min. Also labelled is the neighbouring SrRu_2_O_6_ (100) structural peak. The dashed horizontal line indicates the time at which the cell begins cooling; *t* = 887 mins. c) Ru magnetic moment in SrRu_2_O_6_ from in situ crystallisation experiments (with reaction temperature of 140 °C, 160 °C, 170 °C and 180 °C) as a function of cell temperature during reaction and cooling. The black lines in a) and c) represent values obtained from a powder sample of SrRu_2_O_6_ measured in a separate experiment plotted against the measured temperature at the reaction cell.^[^ ^12^, ^52^ ^]^ d) Ru magnetic moment shown as a function of time, whilst the reaction cell was at reaction temperature (*i*.*e*., cooling data omitted). Error bars in a), c) and d) are 1*σ*.

Refined lattice parameters from SrRu_2_O_6_ obtained from fits against in situ neutron diffraction agree closely with those previously determined from a separate variable‐temperature experiment using a powder sample on the WISH diffractometer at ISIS,^[^ ^12^, ^52^ ^]^ Figure 4a. The *a*‐axis is almost invariant with temperature, owing to a magnetostrictive effect, from strong magnetoelastic coupling of Ru(V) centres, whilst the *c*‐axis reduces as the hydrothermal reaction cell is cooled due to large thermal expansivity in this crystal direction. The close agreement between the values and trends in refined lattice parameters from the in situ data and those expected from the prior heating experiment on a pre‐prepared sample confirm that the temperatures measured by the thermocouples are representative of the actual temperature of the reaction solution. During crystallisation of SrRu_2_O_6_ the sharpening of its Bragg peaks implies a growth in crystallite domain size, and analysis using the Scherrer method shows the formation of micron‐sized crystallites, as expected for this material,^[^ ^12^ ^]^ with crystallite growth mirroring the extent of crystallisation (Figure S10).

The Ru magnetic moments in SrRu_2_O_6_ order antiferromagnetically in both the *ab*‐plane and *c*‐direction, yielding a magnetic structure with a unit cell doubled in the *c*‐direction. This gives additional magnetic Bragg peaks in the neutron powder diffraction patterns, with the most prominent (*hkl* (101)_mag_) at *Q* ∼1.51 Å^−1^ (*d* ∼4.15 Å). Unusually, SrRu_2_O_6_ remains ordered up to 565 K (292 °C), significantly higher than the synthesis temperature, allowing the magnetic structure evolution to be observed as the phase forms (Figure 4b). At all reaction temperatures, the refined Ru moment in the material measured in situ as it forms in solution was found to be consistent with the Ru moment determined separately from a powder sample,^[^ ^12^, ^52^ ^]^ Figure 4c, including those whilst the reaction cell was cooling, which also shows the expected trend of decreasing moment with increasing temperature, as the Néel temperature is approached. The magnetic form factor of Ru^5+^ determined by Parkinson et al.^[^ ^53^ ^]^ was used in this analysis. Figure 4d shows the Ru magnetic moment as a function of time whilst the cell was at the reaction temperature. In the very early stages of SrRu_2_O_6_ formation, the Ru magnetic moment is larger than would be expected, with a large uncertainty, due to the difficulty in accurately fitting a weak diffraction peak that partly overlaps with diffraction peaks from the transient SrRuO_3_(OD)_2_ phase, however its growth can clearly be seen to follow the growth of the atomic structure (Figure 4b). As SrRuO_3_(OD)_2_ reacts and the amount of SrRu_2_O_6_ increases, the refined Ru moment in SrRu_2_O_6_ reaches a stable value as the signal: noise improves and its value matches the expected value at the experiment temperature. Together, this provides convincing evidence that the magnetic structure of SrRu_2_O_6_ is formed simultaneously with the growth of its crystal structure under the hydrothermal reaction conditions.

A fit to the SrRu_2_O_6_ extent of crystallisation, *α*, was made using the Avrami equation^[^ ^54^ ^]^ in the form:

$$\alpha =A\left(1-e^{-{\left(k\left(t-t_{0}\right)\right)}^{n}}\right)$$

**Figure 5:**
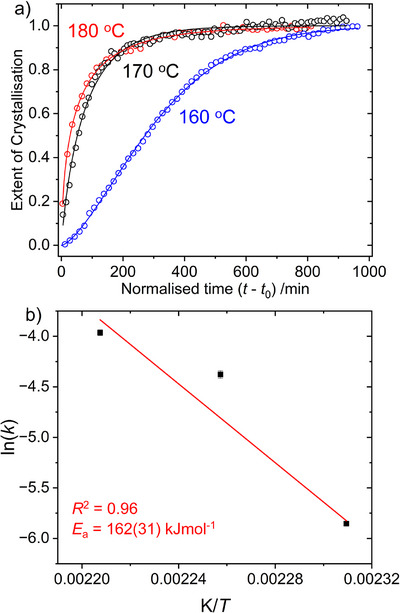
a) Normalised extent of crystallisation of SrRu_2_O_6_ as a function of reaction time during reactions at 160 °C, 170 °C, and 180 °C (data points) with Avrami fits (solid lines). b) An Arrhenius plot for the hydrothermal synthesis of SrRu_2_O_6_ with estimated activation energy (*E*_a_). Error bars correspond to 1*σ*.

Where *A* is a constant, *k* is the rate constant, *t*_0_ is the time at which SrRu_2_O_6_ formation begins and *n* is the Avrami exponent. The expression was fitted to the data using a non‐linear curve fit (Levenberg‐Marquardt algorithm) within the Origin software,^[^ ^55^ ^]^ and the parameters *A*, *k*, *t*_0_, and *n* were allowed to vary freely to optimise the fit to the measured data. The constant was used to produce normalised extent of crystallisation curves, plotted as α versus *t*−*t*_0_, from in situ reactions at 160 °C, 170 °C and 180 °C, as shown in Figure 5a. At 140 °C the reaction was far from complete even after 1100 min (Figure 4a) and an Avrami fit was not possible. The extracted parameters are shown in Table 1.

**Table 1:** Parameters (with 1*σ*) from Avrami kinetic analysis of SrRu_2_O_6_ crystallisation curves.

*T* (°C)	*t*_0_ (min)	*k* (min^−1^)	*n*
160	35.6 ± 4.9	0.00287 ± 0.00041	1.47 ± 0.03
170	68.2 ± 2.0	0.0 1256 ± 0.00049	0.85 ± 0.03
180	75.5 ± 0.1	0.0 1898 ± 0.00056	0.59 ± 0.02

The value of the Avrami exponent changes from < 1 at the two highest temperatures to > 1 at the lowest temperature studied. This would imply a change in mechanism in the solution assembly of SrRu_2_O_6_, and, by analogy to previous application of the model to solid‐state reactions, suggesting a reaction dominated by diffusion control at the higher temperatures.^[^ ^56^ ^]^ Given the rather abrupt change in mechanism over the temperature range studied, an Arrhenius plot can only be used to give an estimate of activation energy, and a value of 162(31) kJ mol^−1^ was determined, Figure 5b. This value, however, can usefully be compared with activation energies determined using the same method from in situ diffraction studies of the hydrothermal crystallisation of oxide materials: for example, a value of 55.10 kJ mol^−1^ for BaTiO_3_,^[^ ^57^ ^]^ 89.0 kJ mol^−1^ for CaTiO_3_,^[^ ^58^ ^]^ and 65 ± 12 kJ mol^−1^ for CoSb_2_O_4_.^[^ ^59^ ^]^ It is conceivable that the higher activation energy found for SrRu_2_O_6_ is due to the influence of the preceding crystallisation of SrRuO_3_(OD)_2_, and indeed Nørby et al. pointed out that when using the same methodology as here for data analysis of crystallisation kinetics that the activation energy of crystallisation involving a phase transformation was around twice the value for the direct hydrothermal crystallisation of a phase.^[^ ^59^ ^]^

## Conclusions

By use of time‐resolved neutron diffraction to follow the hydrothermal crystallisation of the Ru(V)‐containing honeycomb antiferromagnet SrRu_2_O_6_, as well as the disappearance of the Ru(VII) KRuO_4_ starting material, the strontium ruthenate(VI) material, SrRuO_3_(OD)_2_, is seen as a transient phase prior to the formation of the expected product. The presence of this intermediate is prolonged as reaction temperature is lowered from 180 °C to 140 °C, and strongly influences the crystallisation kinetics of SrRu_2_O_6_. At the same time, neutron scattering reveals the growth of the magnetic structure of SrRu_2_O_6_ which we are uniquely able to make from the material because it crystallises below its antiferromagnetic ordering temperature. The refined magnetic moment per Ru seen in situ shows that magnetic structure is formed at the same time as atomic‐scale crystal structure, the first time to our knowledge that this phenomenon has been observed experimentally. Our work highlights the potential for the broader use of neutron scattering for monitoring of crystallisation of materials from null‐scattering reaction cells under realistic conditions to yield information not accessible from X‐ray studies. The methodology should be applicable to study the solvothermal crystallisation of many other families of materials including many oxides and other chalcogenides, silicates and other mineral analogues, and metal‐organic framework materials.

## Conflict of Interests

The authors declare no conflict of interest.

## Supporting information



Supporting Information

## Data Availability

Neutron scattering data are available at the following urls: https://doi.org/10.5286/ISIS.E.RB2220557‐1 https://doi.org/10.5286/ISIS.E.RB1610194‐2 https://doi.ill.fr/10.5291/ILL‐DATA.5‐24‐665
