# Supplementary material for: Stepwise Reduction of Ruthenium and Growth of Magnetic Structure During Hydrothermal Crystallisation of SrRu2O6 from KRuO4
Source: Angew Chem Int Ed Engl. 2025 Nov 17;65(2):e21810. doi: 10.1002/anie.202521810 (PMC12790352; doi:10.1002/anie.202521810)
Supplement: Supplementary file 1 — Supporting Information [file ANIE-65-e21810-s001.pdf]

SUPPORTING INFORMATION

Stepwise Reduction of Ruthenium and Growth of Magnetic Structure During Hydrothermal Crystallisation of $SrRu_2O_6$ from $KRuO_4$

Mark Crossman, Craig I. Hiley, Helen Y. Playford, Ronald I. Smith, Chris M. Goodway, Thomas C. Hansen, and Richard I. Walton

Table of Contents

Page	Description
S2-S3	Experimental detail
S4	Figure S1: View of fully assembled hydrothermal cell
S5	Figure S2: The 'low temperature' furnace
S6	Figure S3: Powder XRD pattern (Cu Kα_1) of $SrRu_2O_6$ prepared in D_2O
S7	Figure S4: Contour maps of *in situ* neutron diffraction (D20, ILL λ = 2.41 Å)
S8	Figure S5: *In situ* neutron diffraction (Polaris Bank 2, ISIS) measured at 170 °C
S9	Figure S6: *In situ* neutron diffraction (Polaris Bank 3, ISIS) measured at 170 °C
S10	Figure S7: *In situ* neutron diffraction (Polaris Bank 4, ISIS) measured at 170 °C
S11	Figure S8: *In situ* neutron diffraction (Polaris Bank 5, ISIS) measured at 170 °C
S12	Figure S9: R_{wp} for the Rietveld fits to powder neutron diffraction patterns
S13	Figure S10: Estimated $SrRu_2O_6$ crystallite size and scale factor with time
S13	References

Experimental Section

Precursors were used as supplied: $KRuO_4$ (STREM Chemicals, 98%), SrO_2 (Sigma-Aldrich) and D_2O (Apollo Scientific, 99.9%). Powder X-ray diffraction data from a preliminary sample made in a 3 ml Teflon-lined autoclave in D_2O were measured using a Panalytical X-Pert Pro MPD in Bragg-Brentano geomentry, using monochromated Cu $K\alpha_1$ incident radiation.

Powder neutron diffraction measurements were carried out on two instruments at two different facilities. Time-of-flight data were collected using Polaris[1] at the ISIS Neutron and Muon Facility, UK which employs 5 banks of ZnS/Ag/^{6}LiF scintillator detectors to access a *d*-spacing range 0.2–30 Å. Constant wavelength data were collected on the D20 diffractometer at the Institut Laue-Langevin, France,[2] which uses a large microstrip position sensitive detector capable of detecting diffracted neutrons covering a scattering range of 153.6° with out-of-plane (ϕ) coverage of 5.8°. The monochromator used consists of aligned crystals of highly-ordered pyrolytic graphite. With a take-off angle of 42° the (002) planes select a wavelength of 2.41 Å and high incident neutron flux ($\sim 4.2 \times 10^7$ s^{-1} cm^{-2}). Neutron diffraction was measured using the hydrothermal cell that is described in the Results and Discussion.

The cell consists of two separate components: the main cell body housing the reaction cavity and a lid. The lid and main cell body are closed using 10 screws through the top of the cell into the bottom portion. A gold O-ring located between circular grooves in the lid and main cell body is compressed during cell assembly to form a pressure-tight seal in the reaction chamber. The cell lid, constructed from steel, also contains two drilled holes that house thermocouples used to monitor the temperature and control the power supply for the external furnace that heats the cell. The cell lid is attached via a Swagelok® fitting to an inline pressure release system. The cell is rated to 250 °C and internal pressures up to 25 bar within the vacuum of the beamline sample chamber.

For the *in situ* measurements on Polaris data were accumulated in 10-minute intervals. On D20 once the shutter is opened diffraction across all angles is measured simultaneously by the position-sensitive detector in ~15 minute accumulations. In both experiments the whole reaction chamber was exposed to the neutron beam, including the very bottom of the cell to record diffraction from all material from within the cell. On Polaris the beam size was 40 mm high x 15 mm wide at the sample position, while on D20 the beamsize was 30 mm high and 10 mm wide.

Analysis of powder neutron diffraction data was carried out with the Rietveld method using the software TOPAS,[3] with a sequential refinement method. The starting model, comprising the three identified phases with arbitrary weights was used for each to avoid bias in fitting each pattern. For time-of-flight data from Polaris, simultaneous fits were carried out against data from banks 2-5 over appropriate time-of-flight ranges (Q_{min} ~1 Å^{-1}, Q_{max} ~ 9 Å^{-1}). For constant wavelength data collected at D20 a specimen displacement and zero error were refined. For all refinements against *in situ* data, atomic coordinates and thermal parameters were fixed to physically reasonable values. Lattice parameters for $KRuO_4$ and $SrRuO_3(OD)_2$ were allowed to deviate a small amount within a small range of expected values to account for thermal expansion. Peak broadening terms for $KRuO_4$ and $SrRuO_3(OD)_2$ were refined against data from the start of an *in situ* experiment and for $SrRu_2O_6$ against data at the end of an *in situ* experiment, and were then fixed for all intermediate collections. A background function (a 25-term Chebyshev polynomial for the constant wavelength data, and a 12-term Chebyshev polynomial for the time-of-flight data), the scale factors (s) of each phase, lattice parameters of $SrRu_2O_6$ and the magnetic moment of the Ru site in $SrRu_2O_6$ were refined. The magnetic form factor of Ru^{5+} determined by Parkinson *et al.*[4] was used. The relative diffracting mass (w) of each phase was then calculated using the equation:

$$w \approx sZMV$$

where Z is the number of formula units, M is the molecular mass of the formula unit and V is the unit cell volume. The relative mass w was chosen instead of phase mass fraction, since w is an absolute measure of the amount of a phase.

In a separate set of refinements against the constant wavelength (D20) data, the $SrRu_2O_6$ peak shape from the final dataset was assumed to be cause by purely instrumental effects (*i.e.* assuming zero strain and infinite crystallite size), and the $SrRu_2O_6$ Scherrer crystallite size was refined (Figure S10).

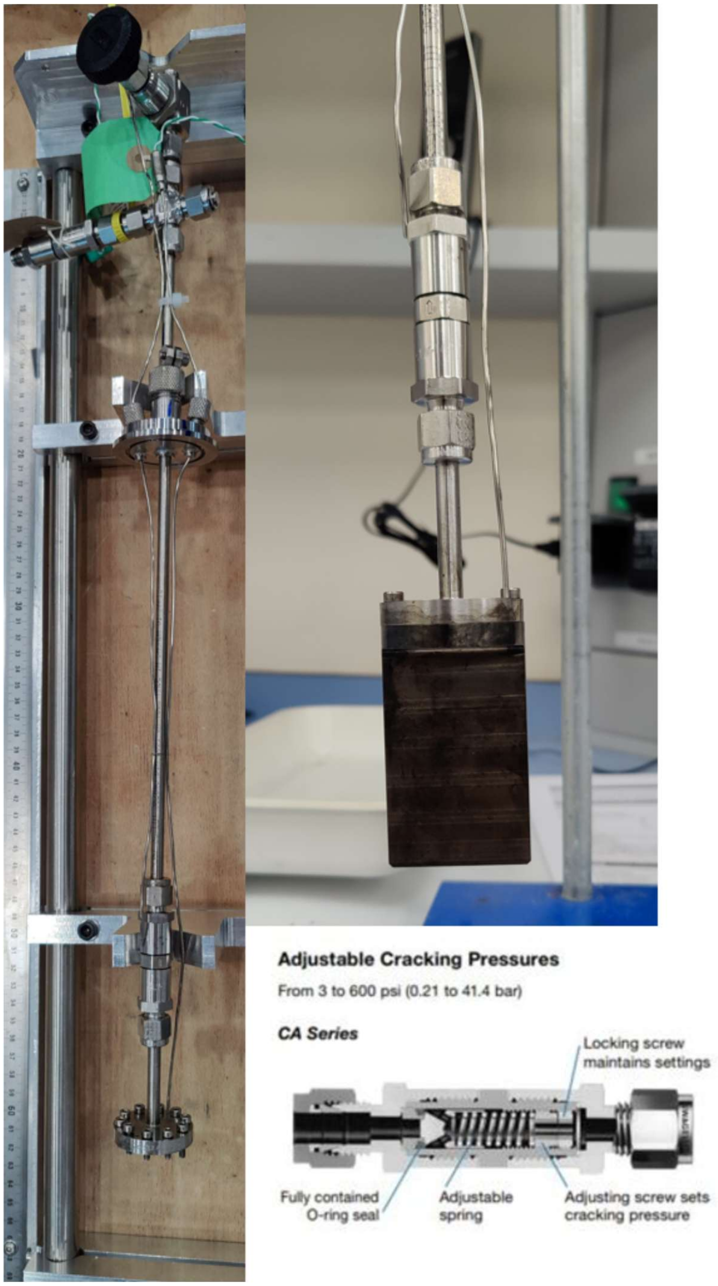

Figure S1: View of fully assembled hydrothermal cell (right) with pressure relief system (left). The lower right panel shows a schematic of the pressure relief system.

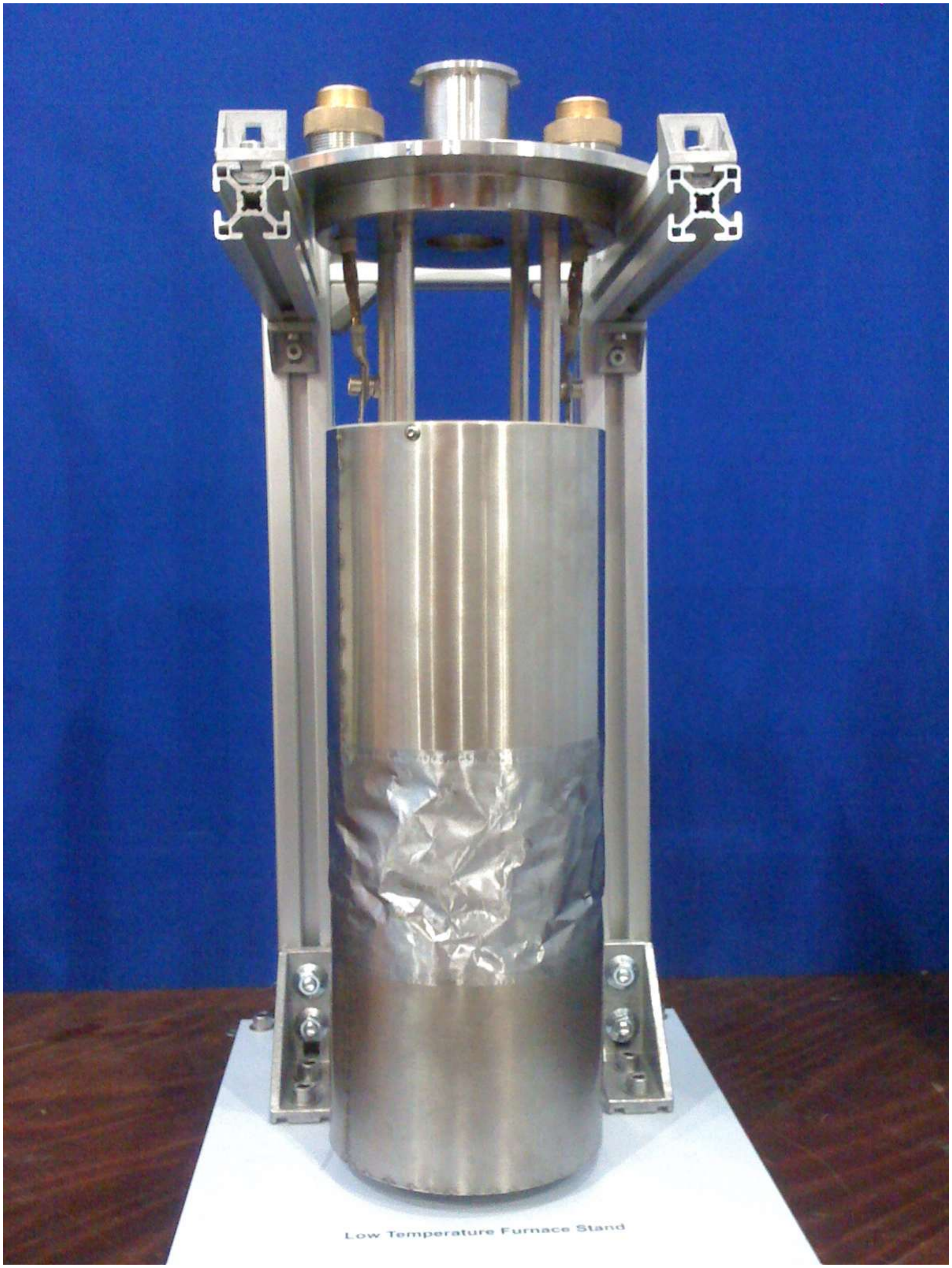

Figure S2: The 'low temperature' furnace used to heat the *in situ* reaction cell.

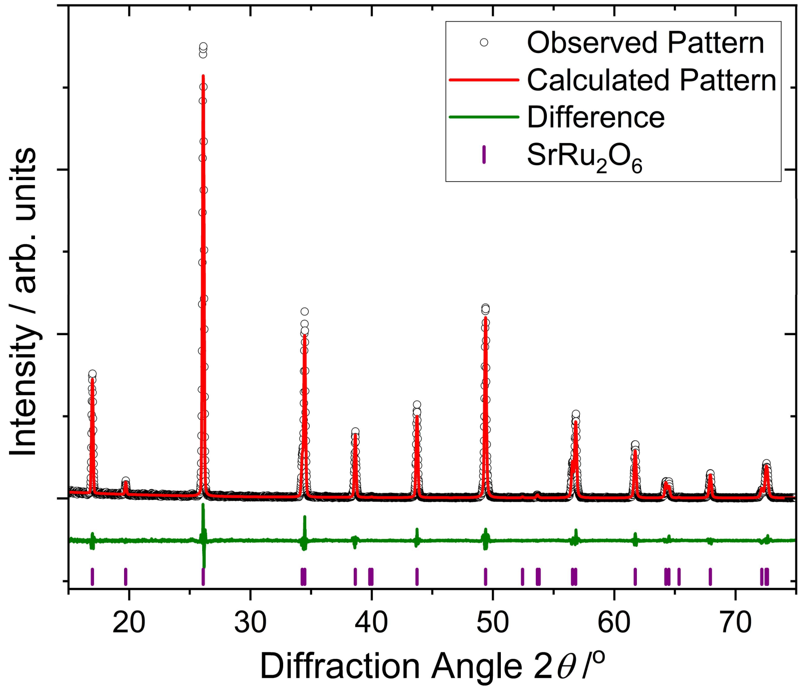

Figure S3: Powder XRD pattern (Cu Kα1) of $SrRu_2O_6$ prepared in D_2O analysed by the Rietveld method. R_{wp} = 14.965 %, $P\bar{3}1m$, *a* = 5.2052(3) Å, *c* = 5.234(7) Å (literature *a* = 5.20460(4) Å, c = 5.23329(7) Å) [5].

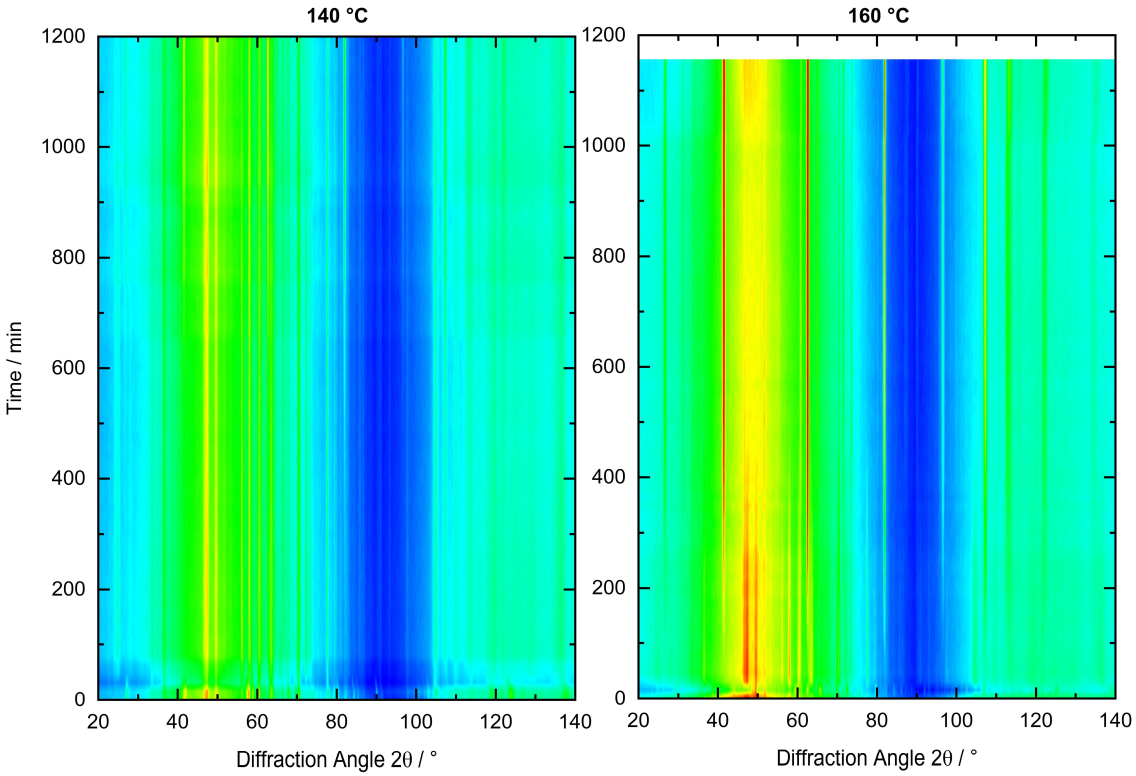

Figure S4: Contour maps of *in situ* neutron diffraction (D20, ILL λ = 2.41 Å) measured at (a) 140 °C and (b) 160 °C from reaction mixture of SrO_2:2$KRuO_4$ in D20 from 0 min to 1200 min (the 160 °C reaction in (b) ends after 1155 min).

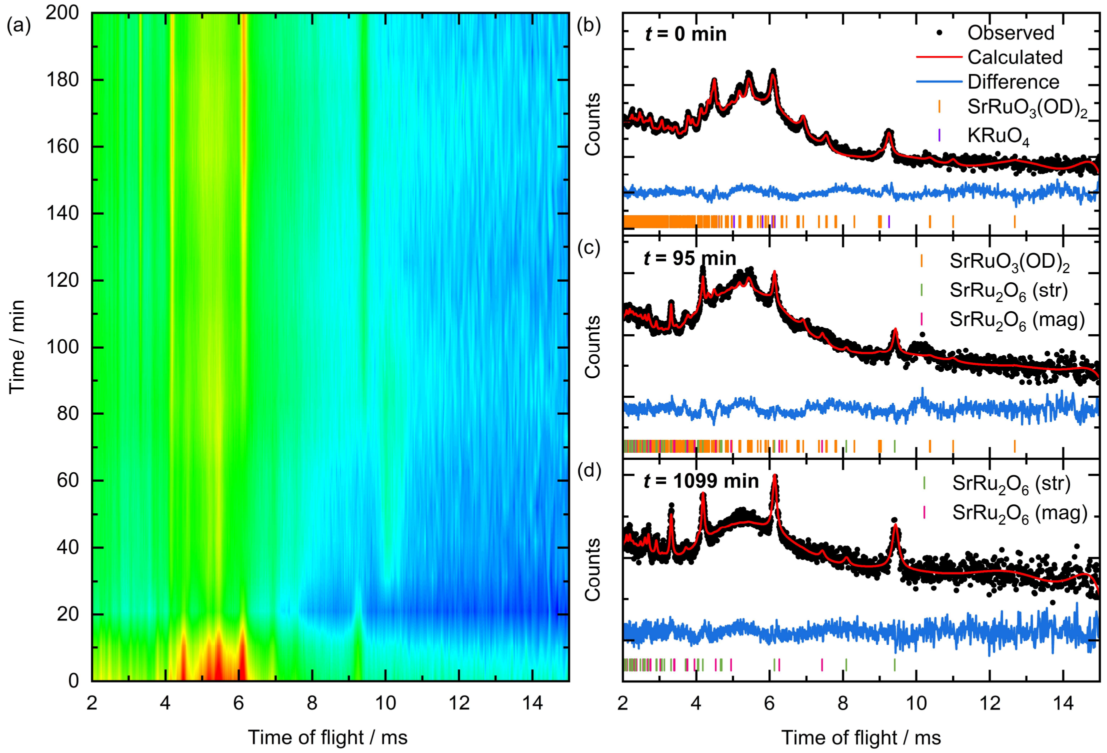

Figure S5: ***In situ*** **neutron diffraction (Polaris Bank 2, ISIS) measured at 170 °C from reaction mixture of SrO_2:2$KRuO_4$ from 0 min to 200 min. (a) Contour map and (b-d) Rietveld fits to individual patterns measured at (b) the start of the reaction (0 min), (c) the point at which the significant amounts of $SrRuO_3(OH)_2$ and $SrRu_2O_6$ are present (95 min) and (d) at the end of the reaction (1099 min).**

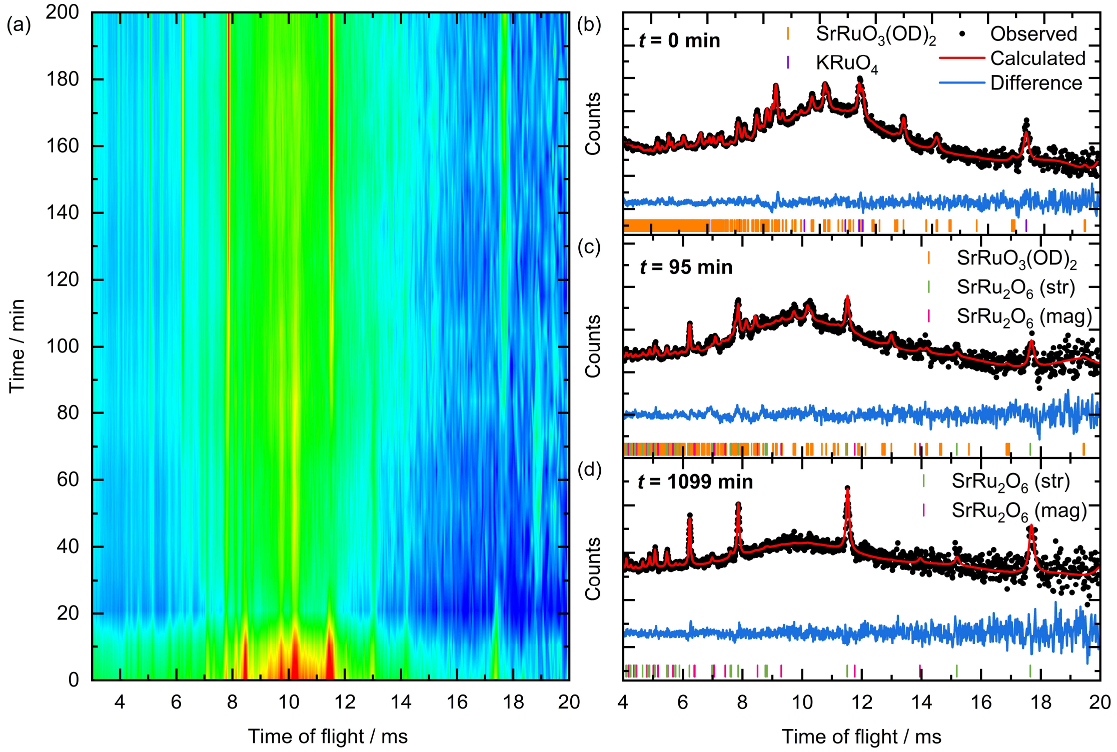

Figure S6: ***In situ*** **neutron diffraction (Polaris Bank 3, ISIS) measured at 170 °C from reaction mixture of SrO_2:2$KRuO_4$ from 0 min to 200 min. (a) Contour map and (b-d) Rietveld fits to individual patterns measured at (b) the start of the reaction (0 min), (c) the point at which the significant amounts of $SrRuO_3(OH)_2$ and $SrRu_2O_6$ are present (95 min) and (d) at the end of the reaction (1099 min).**

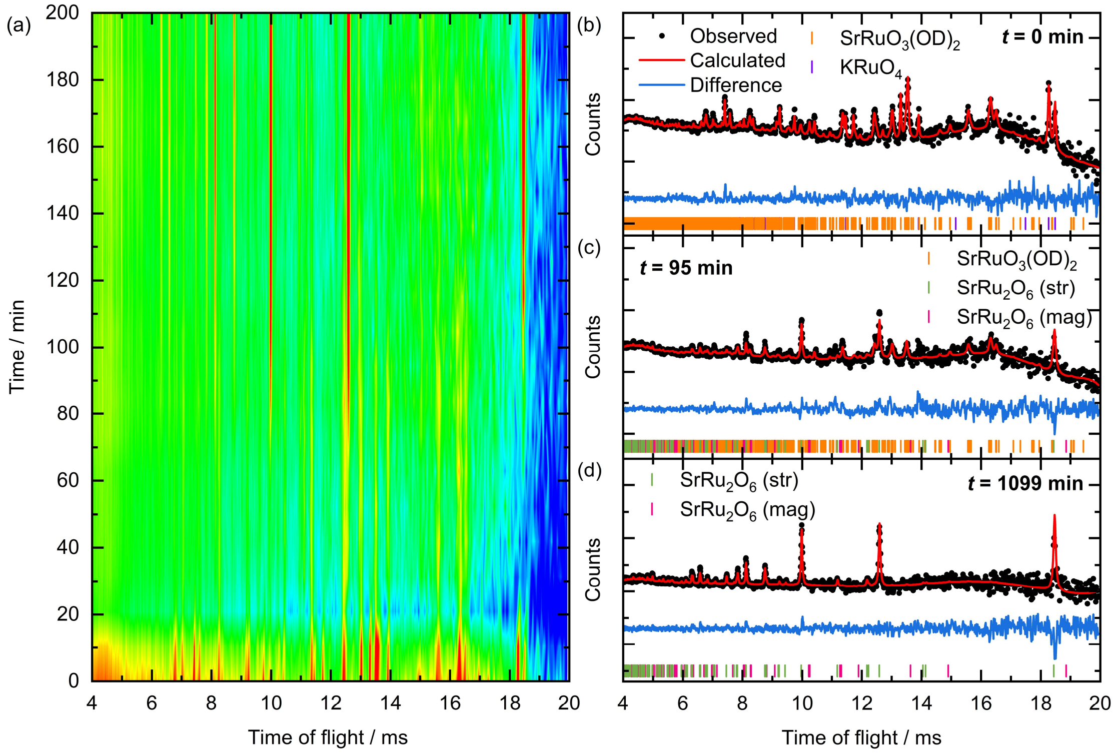

Figure S7: ***In situ*** **neutron diffraction (Polaris Bank 4, ISIS) measured at 170 °C from reaction mixture of SrO_2:$2KRuO_4$ from 0 min to 200 min. (a) Contour map and (b-d) Rietveld fits to individual patterns measured at (b) the start of the reaction (0 min), (c) the point at which the significant amounts of $SrRuO_3(OH)_2$ and $SrRu_2O_6$ are present (95 min) and (d) at the end of the reaction (1099 min).**

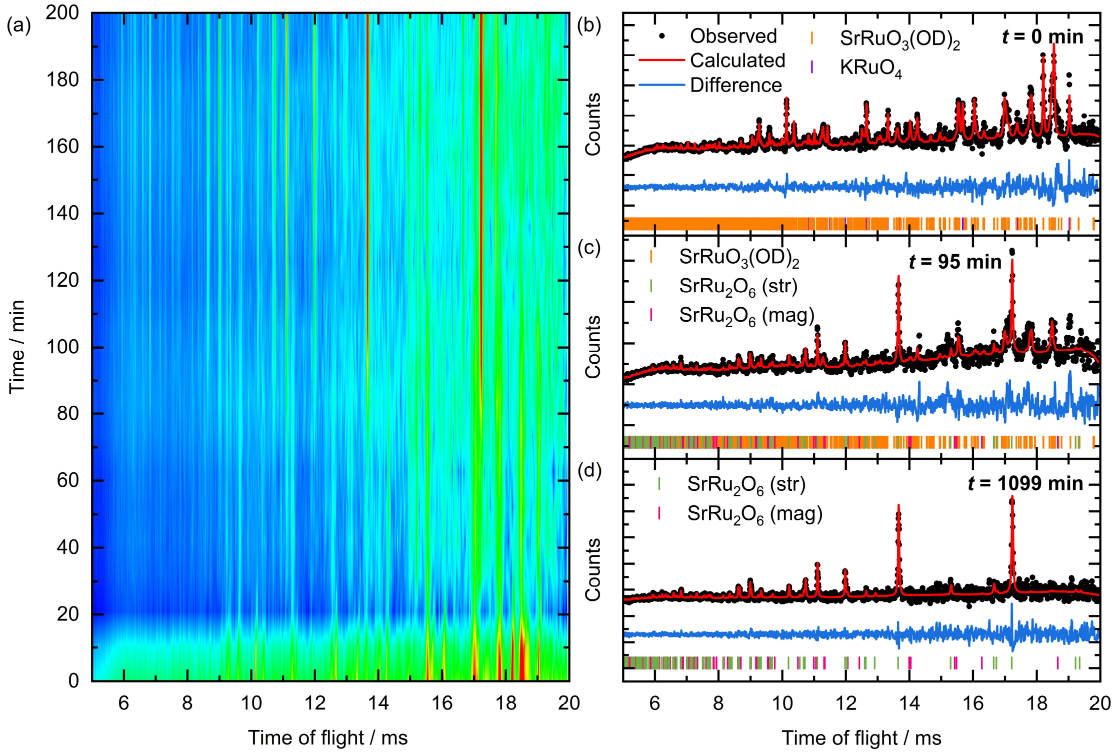

Figure S8: ***In situ*** **neutron diffraction (Polaris Bank 5, ISIS) measured at 170 °C from reaction mixture of SrO_2:2$KRuO_4$ from 0 min to 200 min. (a) Contour map and (b-d) Rietveld fits to individual patterns measured at (b) the start of the reaction (0 min), (c) the point at which the significant amounts of $SrRuO_3(OH)_2$ and $SrRu_2O_6$ are present (95 min) and (d) at the end of the reaction (1099 min).**

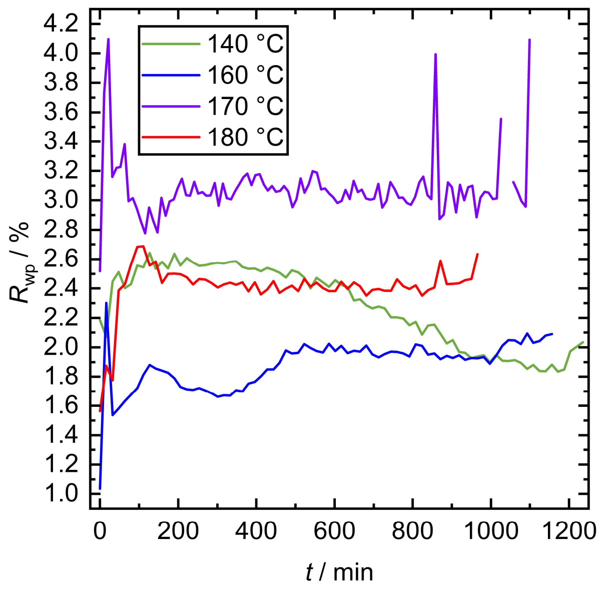

Figure S9. Profile weighted R-factor (R_{wp}) for the Rietveld fits to powder neutron diffraction patterns measured *in situ* at 4 reaction temperatures.

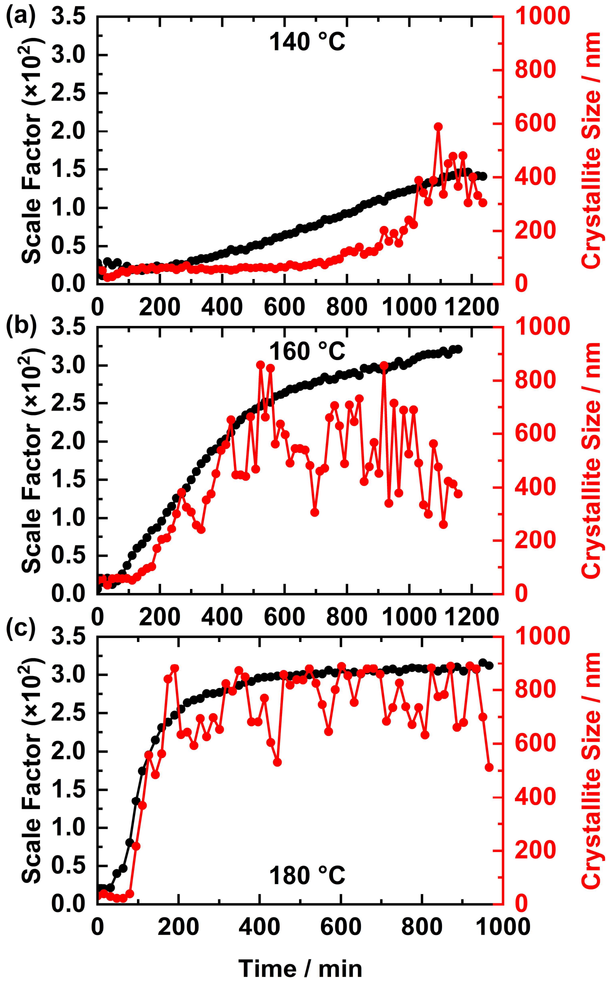

Figure S10. Estimated $SrRu_2O_6$ crystallite size and scale factor developing over time at three reaction temperatures measured on D20 (a) 140 °C, (b) 160 °C and (c) 180 °C.

References

1. Smith, R. I.; Hull, S.; Tucker, M. G.; Playford, H. Y.; McPhail, D. J.; Waller, S. P.; Norberg, S. T., The upgraded Polaris powder diffractometer at the ISIS neutron source. *Rev. Sci. Instrum.* **2019,** *90*.

2. Hansen, T. C.; Henry, P. F.; Fischer, H. E.; Torregrossa, J.; Convert, P., The D20 instrument at the ILL: a versatile high-intensity two-axis neutron diffractometer. *Meas. Sci. Tech.* **2008,** *19*, 034001.

3. Coelho, A. A., TOPAS and TOPAS-Academic: an optimization program integrating computer algebra and crystallographic objects written in C++. *J. Appl. Crystallogr.* **2018,** *51*, 210-218.

4. Parkinson, N. G.; Hatton, P. D.; Howard, J. A. K.; Ritter, C.; Chien, F. Z.; Wu, M.-K., Crystal and magnetic structures of $A_2YRu_{1-x}Cu_xO_6$ with A = Sr, Ba and x = 0.05 to 0.15. *J. Mater. Chem.* **2003,** *13*, 1468-1474.

5. Hiley, C. I.; Scanlon, D. O.; Sokol, A. A.; Woodley, S. M.; Ganose, A. M.; Sangiao, S.; De Teresa, J. M.; Manuel, P.; Khalyavin, D. D.; Walker, M.; Lees, M. R.; Walton, R. I., Antiferromagnetism at *T* >500 K in the layered hexagonal ruthenate $SrRu_2O_6$. *Phys. Rev. B* **2015,** *92*.
